# Condom use increased after a peer group intervention implemented by community volunteers in Malawi

**DOI:** 10.21203/rs.3.rs-3120974/v1

**Published:** 2023-07-04

**Authors:** Kathleen F. Norr, Chimwemwe K. Banda, Cecilia Chang, Shruthi Krishna, Lily C. Kumbani, Li Liu, Linda L. McCreary, Crystal L. Patil

**Affiliations:** College of Nursing, Univiersity of Illinois Chicago; Malawi-Liverpool-Wellcome Trust Clinical Research Programme; School of Public Health, University of Illinois Chicago; School of Public Health, University of Illinois Chicago; Kamusu University of Health Sciences; School of Public Health, University of Illinois Chicago; College of Nursing, Univiersity of Illinois Chicago; College of Nursing, Univiersity of Illinois Chicago

**Keywords:** HIV prevention, condom use, community implementation, Malawi, rural

## Abstract

**Background:**

HIV prevention remains a global priority, especially in sub-Saharan Africa. Our research team previously developed an evidence-based peer group program for HIV prevention called *Mzake ndi Mzake* (Friend to Friend). A community-engaged collaboration adapted the program for community ownership and implementation. Here we report whether this HIV prevention program, implemented by community volunteers, increased condom use among sexually active individuals in rural Malawi.

**Methods:**

Three communities sequentially rolled out the program. Effectiveness was evaluated using repeated surveys. At Time 1, no community had offered the intervention. At Time 2, the first community had offered the intervention and two had not (control group). At Time 3, two communities had offered the intervention and one had not (control group). We used two condom use indicators; condom use frequency in the last 2 months (N = 776) and condom use at last sex (N = 880). For each indicator, the sample included all sexually active persons answering that question at one or more time points. Regression analyses were used to model the two condom indicators over time, controlling for demographic factors, UNAIDS HIV knowledge, safer sex self-efficacy and partner communication.

**Results:**

This peer group intervention implemented by trained community volunteers increased both condom use indicators at Times 2 and 3. In the final regression models. Other predictors of greater condom use for at least one condom use indicator included younger age group (13–19), male gender, not married/cohabiting, high involvement in religiously affiliated activities, higher safer sex self-efficacy, and greater partner communication. Although the intervention increased UNAIDS HIV knowledge, knowledge did not predict condom use.

**Conclusions:**

This community engaged implementation study found that an evidence-based peer group program for HIV prevention increased condom use when delivered by trained community volunteers. Leveraging community strengths and human capital resources facilitated implementation of this effective HIV prevention program in rural Malawi. Community ownership and program delivery by trained local volunteers offers an innovative and cost-effective strategy to address ongoing HIV prevention needs without overburdening heathcare systems in sub-

**Trial registration:**

Clinical Trials.gov NCT02765659 Registered May 6, 2016

## Background

Despite global declines in new Human Immunodeficiency Virus (HIV) infections, 1.5 million people are newly infected per year [1]. Achieving the UNAID goal of reducing new HIV infections to near-zero by 2030 now seems unlikely. Even if this goal is achieved, HIV will remain an endemic health problem for decades, necessitating the continuation of primary HIV prevention programs [2]. Sub-Saharan Africa, despite having the greatest decline in new infections, remains the region the most heavily affected by HIV. The region reports 860,000 new infections annually and a significant gender gap, with 63% of new infections occurring among women [1, 3]. In countries outside of the region, 94% of new infections occur among key populations (e.g., sex workers, men who have sex with men, injecting drug users, transgender persons and their partners) [1]. In sub-Saharan Africa, 49% of new HIV infections occur in the general population [1], creating the need forrobust community HIV prevention programs for the general population as well as targeted prevention for higher-risk key populations.

This study took place in Malawi, a country in Southern Africa which ranks ninth globally in HIV prevalence [4]. The most recent national survey reported 20,000 annual new adult infections with an incidence rate of 0.21% in 2020–21 [5]. Notably, women experienced twice as many new infections as men. Moreover, the southern region of Malawi, where this study was conducted, had the highest burden of HIV [6].

Despite the introduction of newer strategies such as male circumcision and pre-exposure prophylaxis (PrEP), condom use remains a crucial component of combination HIV prevention [7]. Condoms are highly effective, safe, easy to store and use, and cost-effective for individuals and the health system [8–11]. These advantages make condoms ideal for prevention in the general population [5, 7]. Yet condom use remains low in most of sub-Saharan Africa, including Malawi. Only 18% of all adults 15 and older in Malawi reported using a condom the last time they had sex [12]. Although exceptions exist, such as a recent survey in South Africa where 72% of black youth (ages 18–24) reported using a condom at last sex [13], rates of ondom use in other sub-Saharan African countries also are low. Reanalysis of data for sexually active men only from a nationwide survey in Nigeria found less than 20% used condoms consistently [14], and in Zambia only 41% of urban youth said they used a condom at last sex [15]. Even among university students, whose higher education might be expected to promote condom use, a large meta-analysis found that only 53% used a condom the last time they had sex [16], and two recent surveys in South Africa and Nigeria found fewer than 40% of sexually active youth used condoms consistently [17, 18]. Additionally, complacency about HIV has increased as new HIV infections decline and treatment spreads, leading to a decline in both condom use and funding for condom use and other HIV prevention programs [19–21].

Low rates of condom use across sub-Saharan Africa can be attributed to a variety of contextual factors. Factors include unequal gender norms, concerns about sexual pleasure, negative connotations about trust and commitment, especially within marriage, norms restricting discussion of sexuality among partners and between youth and parents, widespread misconceptions about condoms, poverty and limited educational or job opportunities, lack of privacy for purchasing or obtaining condoms at the clinic, and periodic condom shortages [22–24]. The influence of contextual factors is evident in the personal characteristics that are consistently related to more condom use. National surveys and other studies generally show that condom use is more frequent among men, youth, urban residents, those with more education and/or wealth, and those having sex with non-regular partners (defined as a partner who is not a spouse and/or not cohabiting) [5, 12–14, 26]. Some studies have identified additional factors associated with increased condom use, including peer and parent influences, self-efficacy, HIV status awareness, and access to youth-friendly clinics and condoms [14, 15, 17, 25–28].

Despite barriers to condom use, it is important to recognize that the cultural and socioeconomic context evolves over time, and there is some evidence of changing attitude toward HIV prevention in Malawi and other sub-Saharan African countries. Two of the qualitative studies discussed above also indicated some changes in perspectives, viewing condom use as evidence of commitment and caring for one’s partner by protecting them from HIV infection [22, 23]. Condom use rates may be rising among youth in South Africa [13]. Gender differences may also be shifting; two recent studies among sexually active university students in sub-Saharan Africa found no gender differences in condom use [16, 17],

A substantial body of research has demonstrated that behavior change interventions can effectively reduce risk behaviors associated with HIV transmission and increase condom use, and peer group models have been especially successful [29–36]. However, more recent systematic reviews and meta-analyses yield less consistent evidence regarding effectiveness of behavior change interventions. A meta-analysis focusing on interventions for youth found increased condom use for males only [37]. Another review for young women only in sub-Saharan Africa compared behavioral change interventions, structural change interventions (such as cash transfers for school continuation) and combination interventions. Although at least one study for each type of intervention improved condom use, the only intervention that reduced HIV incidence was a cash transfer program in Malawi [38]. In a systematic review focused on social-cognitive factors, only three of eight studies reported significant effects on condom use [39]. A large systematic review in sub-Saharan Africa found that while cross-sectional studies found a relationship between self-efficacy and condom use among youth, most intervention studies did not find that increased self-efficacy led to increased condom use for youth, and where an intervention effect was found it was often observed among young men only [40]. A three-site study and a large meta-analysis examining multiple constructs of behavioral change models found that these constructs related to greater condom use but explained a relatively small portion of the variation in condom use [41, 42]. Many of these studies recommended that interventions incorporate contextual and environmental factors beyond the individual level, more formative evaluation to tailor the intervention to context, and greater community involvement.

In line with growing emphasis on tailoring, two HIV prevention programs in Malawi incorporated incorporate contextual and environmental factors. The BRIDGES II Program for both men and women, which involved small group discussions, community-based participation, and radio messages, increased AIDS knowledge, self-efficacy and condom use at last sex [43]. The Determined, Resilient, Empowered, AIDS-free, Mentored, and Safe (DREAMS) partnership targeted young women ages 15–24 in 10 sub-Saharan African countries. DREAMS components included “club” meetings for young women, family-strengthening and community awareness programs, and youth-friendly health services [44]. Early results from Kenya, Malawi, and Zambia indicated a reduction in violence against young women, but no effects on condom use [45, 46]. In South Africa and Kenya, the DREAMS program was associated with a decline in HIV incidence, but the decline began before this program was introduced and cannot be attributed to DREAMS. However, in Kenya the program was linked to a reduction in number of lifetime sexual partners and fewer instances of condomless sex [47].

Overall, previous behavior change interventions have yielded mixed results. However, the Mzake ndi Mzake peer group intervention, developed and tested by our team and delivered by trained health workers, has shown consistent efficacy in increasing condom use among rural adults, rural youth, and rural and urban health workers in Malawi [48–51]. Given the broad success of the intervention, the next step was to implement the program more widely, starting in southern Malawi where HIV infections are highest. However, due to health worker shortages and increased demand for HIV testing and treatment [52], delivery of Mzake by health workers is not sustainable. Therefore, in partnership with the community, we decided that trained community volunteers could implement the program, taking responsibility for organizing and implementing Mzake. A previous publication documented that HIV prevention knowledge increased after being in the intervention [53]. The purpose of this analysis is to determine whether the Mzake peer group intervention, when delivered by community volunteers, increased condom use for sexually active community members.

## Methods

### Design

This community-engaged implementation study used a hybrid design to simultaneously examine whether the *Mzake* peer group program could be implemented by community volunteers and if the program would remain effective when delivered by trained community volunteers. The study took place in Phalombe District, located in the high-prevalence southern region in Malawi [6]. The program was rolled out across three communities using a stepped wedge design. The order in which communities began implementation was randomly assigned. Effectiveness was determined using repeated surveys of participants in all 3 communities. The Time 1 survey occurred at baseline before any community started rollout. At Time 2, the first community had completed rollout, and the other two communities served as controls. At Time 3, two communities had rolled out the intervention, while the third community had not yet begun rollout and served as controls. Survey retention rates were very high, over 90% at Times 2 and 3.

The design and methods for this analysis are essentially the same as those of a previously reported study showing the positive impact of the intervention on HIV knowledge [53]. However, because condom use is only relevant for those who are sexually active, the condom use analyses used different samples than the knowledge analysis, which used the total sample. Therefore, we briefly present the key features of the overall study, with greater detail about methods specific to the condom use analysis, including the sample, variables, and analysis plan. Further details on the study’s general design and methods can be found in the HIV knowledge paper and a protocol paper summarizing the conceptual basis and design of the larger study [53, 54].

### Site and Sample

This project took place in Phalombe District in southern Malawi where HIV rates are among the highest in the country. To recruit peer group members, the volunteers held community meetings to explain the program. The recruitment criteria included: resided in the specific community, met the age criterion of either over 19 for adults or between 13–19 for youth,, and agreed to participate in the 6-session intervention when offered in their community and to complete the repeated audio-assisted computerized surveys (ACASI). Those who wanted to participate then gave signed consent or, for minors under age 18, signed joint parent-youth assent. Thus, participants self-selected to be in the program.

This article used two indicators of condom use: condom use frequency in the last two months (hereafter, condom use frequency) and condom use at last sex. Only sexually active persons answered the condom use questions. More participants answered the question about condom use at last sex than the question about condom use frequency over the last two months. The sample for each condom use indicator included only persons who answered that question for at least one of one survey (Times 1–3). For example, a youth who was not sexually active at Time 1 or Time 2 but became sexually active and answered both condom use questions at Time 3, would be included in both condom use samples.

The total sample at baseline included 1009 participants. For frequency of condom use, the total sample used for multivariate regression included 771 participants (76.4% of the total sample). For condom use at last sex, the sample included 880 participants (87.2% of the total sample). For both questions, the number of eligible respondents at each time point varied slightly.

### Intervention

The *Mzake* peer group intervention is based upon a conceptual model that integrates the Primary Health Care model of health worker-community collaboration, Bandura’s social-cognitive learning theory, and cultural tailoring [55–58]. *Mzake* consists of six highly interactive sessions facilitated by two trained community adults (for both adult and youth groups). Groups are homogeneous in gender and age category (adult or youth) to respect cultural norms. Those attending at least five sessions receive certificates and share what they learned with family and friends at a celebration after the sixth session. More details about *Mzake ndi Mzake*, the conceptual model that guides the intervention, and the peer leader training is available in previous publications [53, 54, 58].

### Measures

All variables and their operational measures used in this analysis are defined in Table 1. The outcomes of interest were two condom use indicators. The first question asked, “In the past two months, how often have you used condoms?” (hereafter, condom use frequency). The three response categories of never, sometimes, and always, resulted in an interval level indicator of condom use, which was previously used in the *Mzake* efficacy studies [48–50]. Persons who stated they did not have sex in the last two months were treated as missing. The second indicator was the dichotomous question, “Did you use a condom the last time you had sex?”. There was an option to say that the person had never had sexual intercourse, which was also treated as missing. This question, developed by UNAIDS, is easily understood, widely used, and provides comparative data from different times and places. Because it asks about only the most recent occasion, this question is less subject to recall bias but does not provide any sense of how often condoms are used.

Eight covariates \were considered in the multivariate longitudinal models. Baseline variables included sex (self-identified), age group (youth ages 13–19 or adults over 19), education, and level of involvement in religiously affiliated activities (very involved versus somewhat or less involved). Time-varying covariates included whether married or cohabiting versus single and three psychosocial factors previous research has linked to condom use: UNAIDS comprehensive HIV prevention knowledge (hereafter UNAIDS knowledge), a safer sex self-efficacy index (hereafter self-efficacy) and a safer sex partner communication index (hereafter partner communication). Both the self-efficacy and partner communication measures included several different behaviors and are indices rather than scales.

### Procedures

Implementation used a simple 3-step implementation model: plan, roll out and sustain. During planning, each community’s leaders established a Coordinating Committee. The Committee selected and coordinated training for volunteer peer group facilitators and recruited community members to participate in the *Mzake* program. Then the rollout of the program occurred. In each of the three communities, facilitators offered the peer group program to 12 groups of adults and 16 groups of youth (ages 13–19). After rollout was completed, each community sustained the project at least until the end of grant funding.

### Analysis

For each condom use indicator, intervention versus control group differences were first examined using Chi-squared tests at each time point. Then intervention effectiveness over time for each condom use indicator was evaluated using multivariate models for longitudinal design, controlling for baseline and time-varying demographic and psychosocial factors. Mixed-effects cumulative logit models were employed to model the ordinal question about frequency of condom use. Generalized Estimating Equation models (GEE) were used to model the UNAIDS dichotomous question about condom use at last sex. The number of random effects in the mixed-effects cumulative logit model and variance-covariance structures for repeated measures in GEE were selected using fit statistics such as Akaike Information Criterion (AIC). Significant covariate effects were selected using backward selection methods, controlling for Type I error probability of 0.05. Time trends were evaluated in all regression models using polynomial terms when necessary. Due to the stepped wedge design, in which all individuals were in the control group at baseline, group effect was not entered in the regression models as a main effect. Instead, group by time interaction terms, which indicated group differences over time, were the primary focus of inferences. Covariates-adjusted time-point specific group differences and their 95% confidence intervals from multivariate models were reported for both condom use indicators ( ordinal proportional odds for frequency of condom use and adjusted odds ratios for condom use at last sex). The statistical software program SAS (9.4) was used for all statistical analyses [59].

## Results

### Participant characteristics

Demographic and psychosocial information is presented for the sexually active sample for each question at baseline and at each time point for the time-varying covariates (Table 2). Sample characteristics for this analysis differ somewhat from the total sample reported earlier [49]. Like the total sample, the condom use samples have approximately equal numbers from each group village and roughly equal proportions of males and females. However, many more youth (ages 13–19) than adults were not sexually active, so only around 40% of the condom use samples were youth, compared to 54% of the total sample. Because older youth were more likely to be sexually active, the proportion who attended at least some secondary school was also higher for the condom use samples. The condom use samples also had a substantially higher proportion of persons who were married or cohabiting at baseline than the total sample (64.6% for frequency of condom use and 56% for the sample for condom use at last sex). In contrast, only 43% of the total sample were married or cohabiting. Although the proportion who were married or cohabiting increased over time, the increase was not large. Like the total sample, a majority of participants in both condom use samples reported high involvement in religiously affiliated activities. Mean scores for all three social-psychological covariates increased over time.

### Intervention effects on condom use

In bivariate analyses, both indicators of reported condom use were significantly higher for those in the intervention group than the control group at Time 2 and Time 3 (Table 3). For the ordinal variable asking about the frequency of condom use over the last two months at Time 1 over half (51.3%) of the participants said that in the last 2 months they always used a condom, 26% used sometimes and 22.4% never used a condom. At Time 2, reported condom use by those in the control group declined, while reported condom use in the intervention group remained high; 52.9% reported that they used a condom every time and only 17.4% said that they never used a condom. At Time 3, the significant difference between the intervention and control groups remained, with higher percentages reporting more frequent condom use (always or sometimes) in the intervention group.

Greater use of a condom at last sex by the intervention group compared to the control group was also significant at both Time 2 and Time 3. For condom use at last sex, 59% of the sample reported using a condom at Time 1. At Time 2, the control group reported using a condom less often, while the intervention group reported more use (52.7% vs. 71.4%). At Time 3, the group difference was smaller (55.7% vs. 63.6%), yet still remained statistically significant.

We then evaluated the impact of the intervention on the condom use indicators using multivariate regression models when controlling for significant demographic and social-psychological factors (Table 4). After the model selection process, age, partner status, self-efficacy, and communication between partners were found to be associated with both condom use outcomes. Being adult and being married and/or cohabiting related to less condom use for both indicators. Gender was associated only with condom use in the last two months; women reported less frequent condom use. Involvement in religiously affiliated activities was associated only with condom use at last sex; greater involvement increased the odds of condom use at last sex. Both self-efficacy and partner communication significantly increased condom use.

The effect of time on both condom use indicators was negative, indicated that the probabilities of both condom use indicators decreased steadily over time for the control group. However, the intervention by time interaction was positive for both condom use indicators at Time 2 and at Time 3, indicating that the intervention was successful at reversing the decreasing condom use trend in the population (Table 4). At Time 3, the intervention group was about twice as likely to use condoms frequently in the last two months as the control group (AOR = 2.04, 95% CI 1.24, 3.35), and about 1.8 times more likely to have used a condom at last sex as that control group (AOR = 1.80, 95% CI = 1.12, 2.89).

## Discussion

This community-based peer group intervention delivered by trained community volunteers increased the frequency of condom use over the last two months and condom use at last sex. These findings, coupled with the previous finding of increased HIV prevention knowledge [54], indicate sustained effectiveness of the Mzake program when implemented by community volunteers.

In addition to the impact of the Mzake program on condom use, our multivariate analyses also identified four covariates that related to both frequency of condom use in the past two months and condom use at last sex: age, relationship status, self-efficacy and partner communication. Consistent with prior studies [5, 12–14, 27], being a youth and not having a regular partner were associated with increased condom use.

Higher safer-sex self-efficacy and partner communication also were significant predictors for both condom use variables. These factors are fundamental to the social-cognitive conceptual model that underpins Mzake as well as other peer group interventions. Four studies of youth reported results that were consistent with our findings showing that greater self-efficacy related positively to condom use [17, 18, 33, 41]. However, two systematic reviews of interventions and two additional studies of youth in sub-Saharan Africa had different findings. Although there was often a positive bivariate relationship between self-efficacy and condom use, in multivariate analyses, self-efficacy did not lead to increased condom use or led to greater condom use for young men only [16, 27, 40, 43]. Fewer studies examined partner communication. Three studies reported that partner communication related to greater condom use [15, 18, 27], while another found that partner communication did not relate to condom use in bivariate or multivariate analyses, [17], Our finding demonstrating positive associations of self-efficacy and partner communication with greater condom use contributes to the growing body of evidence supporting the effectiveness of the social-cognitive learning model in facilitating behavior change. However, contrary findings highlight the need for more research to understand the complex relationships among self-efficacy and partner communication, gender and gender-based inequities, and HIV prevention behaviors such as condom use,

Being a man was associated with more frequent condom use over the last two months but did not relate to using a condom at last sex. Congruent with these findings, a large meta-analysis and a survey of university students also found no difference in condom use between men and women [16, 17]. However, our findings contradict many other studies where condom use has been higher for men than women across sub-Saharan Africa regardless of how condom use was measured [5, 12, 13, 18, 27, 37, 40].

Level of involvement in religiously affiliated activities was positively related to use of a condom use at last sex but not to condom use frequency over the last two months. Two other studies examined religious affiliation and condom use [26, 27], but no other studies were located that examined involvement in religiously affiliated activities. Historically, the majority of messages disseminated by faith-based leaders and organizations have opposed condom use. However, there is also evidence that some faith-based leaders and organizations actively contribute to HIV and AIDS initiatives and are receptive to supporting HIV prevention programs as part of their faith-based mission [60–62]. Changing perspectives of religious leaders and members about HIV prevention and condom use may relate to this study’s finding of a positive relationship between greater involvement in religious activities and use of a condom at last sexual intercourse. The influence of religious leaders and organizations across sub-Saharan Africa is substantial. One small piece of evidence supporting their continuing importance is that nearly two-thirds of this study’s participants described themselves as highly involved in religiously affiliated activities such as choir, sports, charitable activities, and prayer meetings. A deeper understanding of how religious factors affect condom use and how this is changing over time is very important to increase effectiveness of HIV prevention interventions.

Two factors, education level and UNAIDS HIV prevention knowledge, are worth noting because they did not show a significant relationship with our indicators of condom use and were therefore excluded from the final regression models. In large surveys, higher levels of education were associated with increased condom use [5, 12, 14]. However, two studies found results similar to ours, that higher education did not predict greater condom use in multivariate analysis [15, 26].

Although our previous publication showed a substantial increase in UNAIDS HIV knowledge with the Mzake program [53], this increased knowledge was not a predictor of increased condom use in multivariate analyses. Our results are similar to a survey in South Africa and an intervention study in Malawi [13, 43]. However, several cross-sectional analyses using national demographic and health surveys in five African countries, as well as one systematic review, reported a positive relationship between HIV prevention knowledge and greater condom use [14, 25, 28, 34]. One of these studies examined married persons with an extramarital partner and found that high HIV knowledge related to more condom use with the extramarital partner for men but not women [28]. These mixed results suggest that knowledge about HIV transmission is important for HIV prevention, but knowledge alone may not be sufficient to change behaviors. This pattern of findings is congruent with social-cognitive learning and other behavioral change theories, which posit that other factors, such as self-efficacy and partner communication, are more directly tied to changing behaviors. Therefore, when knowledge is included in multivariate analyses along with factors like self-efficacy and partner communication, the impact of HIV knowledge may be diminished.

One question that emerges from these results is why the Mzake program has been effective when other similar interventions have not reported similar results. Several factors have been identified by both the research team and community volunteers as potential contributors to Mzake’s effectiveness. In these communities, HIV prevention was a top priority, and the people had strong enthusiasm for implementing an HIV prevention program that had demonstrated success in Malawi. The program was offered to community residents above age 12 regardless of age, gender, or social position, so youth and parents, male and female partners, all types of leaders and ordinary people, participated in the same HIV prevention program and could support each other. Community members in our previous efficacy studies noted how important mutual support was in helping people change behaviors. Throughout the implementation process, a community-engaged approach was used, which has been shown to increase equitable and effective program implementation [62]. Drawing on their in-depth understanding of the local context, community members actively participated in decision-making processes, including selection and training of peer group facilitators, determining suitable locations and meeting times, and maintain program records. The research team provided initial training for volunteers and evaluated effectiveness. Moreover, extensive formative evaluations were carried out during initial efficacy studies and for this community implementation program. This strategy fostered retention of Mzake’s core components while allowing for adaptations needed to suit the context. This community-engaged program is congruent with previous researchers’ recommendations that contextual factors at the societal and community levels and formative evaluation to guide adaptation can improve the implementation process and program effectiveness [39–42].

### Limitations

A major limitation, but also likely a strength, of this study, is that neither the community volunteers who delivered the program nor the program participants were randomly selected. Community leaders decided who would serve to coordinate the Mzake program, based largely on their prior volunteer work for the community and proven reliability. This committee then chose individuals who would become peer group facilitators. Selection was based on prior knowledge of who had a proven record of community service and well as adequate literacy to use the manual effectively. Similarly, community members were invited to an open community meeting, where they made the decision to participate. While the non-random selection of individuals introduces biases, allowing community autonomy in program implementation is an important strategy for supporting a community-delivered HIV prevention program. Another limitation is the repeated use of the same survey at each evaluation time point. This practice may have influenced reporting of condom use independent of the effect of the intervention because it may have prompted a socially desirable response that inflated the reporting of condom use. Social desirability cannot be ruled out as a factor in the intervention group, but neither condom use indicator increased greatly in the control group.

## Implications

Preventing new HIV infections remains a pressing priority in sub-Saharan Africa. HIV prevention is an essential part of controlling the epidemic [2]. This community-based implementation study found that when an evidence-based peer group program for HIV prevention was organized and delivered by trained community volunteers, the program significantly increased condom use as well as HIV prevention knowledge [53]. These findings provide valuable evidence that community volunteers can effectively implement peer group programs that result in increased HIV prevention behaviors. This approach is especially pertinent in the face of new challenges including growing complacency toward HIV, declining HIV prevention funding, and decreasing condom use as well as persistent health worker shortages [19–21, 52]. Leveraging community strengths and human capital resources facilitated implementation of this effective HIV prevention program in southern Malawi. This community engaged approach, where the community owns the program which is delivered by trained local volunteers, offers an innovative and cost-effective strategy to address ongoing HIV prevention needs without overburdening heathcare systems in sub-Saharan Africa.

## Figures and Tables

**Table 1:** Condom use measures and covariates

Variable	Operational Measures

**Outcomes**	

Condom use frequency in the last 2 months	In the last two months, how often did you use a condom? (Scored 3 = Always, 2 = Sometimes, 3 = Never)

Condom use at last sex	Now think about the most recent time you had sexual relations. Did you use a condom? (Scored 1 = Yes, 2 = No).

**Covariates**	

Demographic factors	• Age group (adult > 19 versus youth ages 13–19)
	• Sex
	• Education (did not complete primary school, completed primary school, completed secondary school)
	• Partner status (married and/or cohabiting versus single)
	• How involved are you in activities related to your church or mosque? (Scored 1 = very involved, 0 = somewhat, seldom or never involved)

Psychosocial factors	• UNAIDS Comprehensive HIV prevention knowledge. Defined as correctly answering **all 5 True/False items.** Score 1 = all 5 items correct, 0 = one or more questions incorrect. Items include: 1) Can the risk of HIV transmission be reduced by having sex with only one uninfected partner who has no other partners?; 2) Can a person reduce the risk of getting HIV by using a condom every time they have sex?; 3) Can a healthy-looking person have HIV?; 4) Can people get the AIDS virus from mosquito bites?; 5) Can a person get the AIDs virus by sharing food with a person who has AIDS?.
	• Safer sex self-efficacy index (4 items related to HIV prevention) How confident are you that you can: Get your partner to agree to using a condom?; Avoid having sex if you have decided to abstain? Refuse to have sex if a condom is not going to be used?, Have an HIV test with partner; score was the sum of responses, 3 = very confident, 2 = somewhat confident. and 1 = not confident, Total score = sum of all 4 items; Range: 4–12)
	• Partner communication index (4 items related to HIV prevention). In the last 2 months have you talked with a partner about: Not having sex with each other?; Using condoms every time the two of you have sex?; can have an HIV test with your partner?, Being faithful to each other); Having an HIV test together; Total score = sum of items answered “Yes”, Range 0–4)

**Table 2:** Demographic and psychosocial factors for the condom use indicators

	Condom use frequency in the last two months (Baseline N = 567)	Condom use at last sex (Baseline N = 749)
**Baseline factors:**		
**Community, N (%)**		
Community 1	204 (36.0%)	260 (34.7%)
Community 2	177 (31.2%)	246 (32.8%)
Community 3	186 (32.8%)	243 (32.4%)
**Gender, N (%)**		
Female	262 (46.2%)	358 (47.8%)
**Age Group**		
Adult	347 (61.2%)	442 (59.0%)
**Education, N (%)**		
Did not complete primary school	253 (44.6%)	336 (44.9%)
Completed primary school	213 (37.6%)	289 (38.6%)
Completed secondary school	101 (17.8%)	124 (16.6%)
**Involvement in religiously affiliated activities, N (%)**		
Very involved	366 (64.6%)	477 (63.7%)
**Time-varying covariates:**		
**Partner Status, N (% married or cohabiting)**		
T1	355 (62.6%) N = 567	420 (56.1%) N = 749
T2	331 (61.9%) N = 535	386 (54.7%) N = 706
T3	390 (68.9%) N = 566	446 (58.8%) N = 758
**UNAIDS Knowledge, N (%)**		
T1	267 (47.1%) N = 567	336 (44.9%) N = 749
T2	296 (55.3%) N = 535	374 (53.0%) N = 706
T3	323 (57.0%) N = 567	432 (57.0%) N = 758
**Safer sex self-efficacy Index, Mean (SD)**		
T1	10.96 (1.44) N = 567	10.91 (1.49) N = 749
T2	11.02 (1.35) N = 534	11.01 (1.36) N = 705
T3	11.05 (1.36) N = 567	11.06 (1.32) N = 758
**Partner communication index, Mean (SD)**		
T1	2.81 (1.13) N = 460	2.81 (1.12) N = 540
T2	2.89 (1.10) N = 472	2.89 (1.12) N = 567
T3	2.95 (1.07) N = 521	2.92 (1.11) N = 618

**Table 3:** Bivariate group differences in condom use indicators over time

	Survey 1		Survey 2		Survey 3	
**A. Condom use frequency in the last 2 months, N (%)**						
	**Control** **(N = 567)**	**Intervention**	**Control** **(N = 363)**	**Intervention** **(N = 172)**	**Control** **(N = 186)**	**Intervention** **(N = 381)**
**Never**	127 (22.4%)	-	108 (29.8%)	30 (17.4%)***	53 (28.5%)	68 (17.9%)*
**Sometimes:**	149 (26.3%)	-	120 (33.1%)	51 (20.7%)	57 (30.7%)	134 (35.2%)
**Always:**	291 (51.3%)	-	135 (37.2%)	91 (52.9%)	76 (40.9%)	179 (47.0%)
**B. Condom use at last sex, N (%)**						
	**Control** **(N = 749)**	**Intervention**	**Control** **(N = 486)**	**Intervention** **(N = 220)**	**Control** **(N = 244)**	**Intervention** **(N = 514)**
**No:**	307 (41.0%)	-	230 (47.3%)	63 (28.6%)***	108 (44.3%)	187 (36.4%)*
**Yes:**	442 (59.0%)	-	256 (52.7%)	157 (71.4%)	136 (55.7%)	327 (63.6%)

Significance level, control and intervention groups difference:

* p < 0.05

** p < 0.01

*** p < 0.001

**Table 4:** Multivariate Model Results for Intervention effects on condom use Times 1–3

	Condom use frequency in the last two months (N = 771)		Condom use at last sex (N = 880)	
	Estimate (SE)	OR (95% CI)	Estimate (SE)	OR (95% CI)
Age Group:				
Adult	−0.56(0.16)***	0.57 (0.42, 0.78)	−0.57 (0.15)***	0.56 (0.42, 0.76)
Partner Status:				
Married/cohabiting	−1.02 (0.17)***	0.36 (0.26, 0.50)	−0.53 (0.15)***	0.59 (0.44, 0.79)
Gender:				
Female	−0.39 (0.13)**	0.68 (0.53, 0.87)		
Involvement in religiously affiliated activities:				
Very involved			0.34 (0.13)**	1.41 (1.10, 1.81)
Safer sex self-efficacy	0.11 (0.04)**	1.12 (1.03, 1.22)	0.12 (0.04)**	1.12 (1.04, 1.22)
Partner communication	0.76 (0.06)***	2.15 (1.91, 2.41)	0.54 (0.05)***	1.71 (1.54, 1.90)
Time	−0.25 (0.08)**	-	−0.13 (0.08)	-
**Time** * **Intervention**	**0.23 (0.08)** **	**-**	**0.20 (0.08)** *	**-**
Intervention-control difference, Survey 2: OR (95% CI)	1.59 (1.15, 2.21)		1.48 (1.08, 2.03)	
Intervention-control difference, Survey 3: OR (95% CI)	2.01 (1.23, 3.30)		1.81 (1.13, 2.90)	

Significance level control and intervention groups difference:

* < .05

** < .01

*** < .001

## Data Availability

The datasets generated and/or analyzed during the current study are not yet publicly available but will be made available based on reasonable request by emailing the corresponding author.

## Abbreviations

HIV: Human immunodeficiency virus
PMTCT: Prevention of mother to child transmission
UNAIDS: Joint United Nations Programme on HIV/AID
